# Low-frequency ultrasound treatment reduces susceptibility to *Salmonella* infection in aged mice

**DOI:** 10.1128/jb.00176-25

**Published:** 2025-08-12

**Authors:** Parisa Zangoui, Ekta Singh, Michael P. Sheetz, Linda J. Kenney

**Affiliations:** 1 Department of Biochemistry and Molecular Biology, University of Texas Medical Branch 12338, Galveston, Texas, USA; 2 Sealy Center for Structural Biology, University of Texas Medical Branch 12338, Galveston, Texas, USA; University of Virginia School of Medicine, Charlottesville, Virginia, USA

**Keywords:** low-frequency ultrasound, *Salmonella*, aging, immune response, ICAM-1, SDF-1, KC/CXCL1

## Abstract

Increased susceptibility of elderly populations to infection is a major concern, as recently experienced by the coronavirus pandemic. Therefore, the treatment of infected elderly individuals is of immediate interest. Here, we report the use of ultrasound treatment to reduce *Salmonella* infection in elderly individuals. *Salmonella enterica* serovar Typhimurium enters the human body through the ingestion of contaminated food or water and causes gastroenteritis. The Centers for Disease Control (CDC) reports 420 deaths per year in the United States caused by the *Salmonella* pathogen, especially in the elderly population. In this study, we investigated the susceptibility to *Salmonella* infection in young and old mice by enumerating the number of viable bacteria in the liver 3 days post-infection. We then explored the effect of ultrasound treatment (low-frequency ultrasound [LFU]) prior to infecting aged mice with *Salmonella*. We observed a higher colonization of *Salmonella* infection in the livers of old mice compared to young mice. Colonization was significantly reduced when old mice were treated with LFU prior to infection, which significantly reduced colonization. Chemokine analysis revealed a significant increase in ICAM-1, SDF-1, and KC/CXCL1 in aged, treated mice. Ultrasound treatment rejuvenated the immune system in old mice, reducing their susceptibility to *Salmonella* infection.

**IMPORTANCE:**

Our study demonstrates the efficacy of low-frequency ultrasound (LFU) treatment in significantly rejuvenating the immune system in aged mice and reducing their susceptibility to *Salmonella* infection. These findings underscore the potential of LFU as a therapeutic intervention to boost immune function in elderly populations, reducing the risk of infectious diseases.

## INTRODUCTION

It is well known that elderly populations are at greater risk of succumbing to infectious diseases. Higher mortality rates of aged mice with *Salmonella* were reported in 1972 (1), and enhanced levels of *Salmonella* were observed in the livers and spleens of aged rats (2). Colonization of *Salmonella* in the ileum, colon, Peyer’s Patch, liver, and spleen of aged mice was higher than in young mice (3). The increased susceptibility to infection can be due to a decline in the immune response, aging in organs or organ senescence, additional disease, or environmental factors such as malnutrition (4). A clear understanding of the impact of aging on susceptibility to infection requires a better understanding of aging mechanisms.

Aging or cellular senescence was first described by Hayflick as a cell proliferation arrest in an aging culture of human fibroblasts (5). Cellular senescence is irreversible cell cycle arrest resulting from various stress conditions such as ionizing radiation, oxidative stress, UV exposure, genotoxic agents, and telomere attrition (6). One of the hallmarks of senescent cells is a secretion of a cocktail of cytokines, chemokines, growth factors, proteases, and angiogenic factors known as senescence-associated secretory phenotype, or SASP (6). Secretion of SASP factors results in inflammatory aging and drives immune senescence in both innate and adaptive immunity (7, 8). In aged mice, greater susceptibility to *Salmonella* infection has been associated with lower levels of TNF-α and IFN-γ from the spleen and mesenteric lymph node cells due to reduced neutrophils and macrophages upon infection (3).

The higher number of deaths by *Salmonella* Typhimurium in elderly individuals and the complexity of aging mechanisms highlight a need for the development of anti-aging tools to protect older people against infection. In recent years, various senolytic approaches have been implemented by genetically or chemically targeting senescent cells (9, 10). Physical exercise (11) and immune rejuvenation using young mice spleen cells (1) in aged mice have been applied as an approach to protect the elderly from *Salmonella* infection.

Another approach to rejuvenating aged mice is the application of low-frequency ultrasound (LFU). Low-frequency sonophoresis (LFS) refers to sound waves in the frequency range of 20–200 kHz that can penetrate tissues with thermal, mechanical, and cavitation effects. LFS usage dates to the 1950s for the treatment of arthritis. The medical applications of LFS (range: 20–200 kHz) include transdermal drug delivery, promotion of tissue healing, thrombolytic effects, and antitumor effects (12). Previously, we used LFU in the apoptosis of tumor cells (13) and *in vitro* and *in vivo* rejuvenation of chemically induced and replicative senescent cell lines, as well as aged mice (14). Treatment of aged mice with LFU was able to rejuvenate them in terms of physical activity, and LFU treatment increased lifespan (14). More recently, mice fed a high-fat, high-sucrose diet combined with streptozotocin developed insulin resistance and diabetes, which was modified by LFU treatment (15). Interestingly, the transcriptomic analysis indicated that LFU primarily reduced inflammatory and immune-related gene expression, potentially by promoting a shift toward an anti-inflammatory (M2) macrophage profile (15). Thus, it is logical that LFU could aid in reversing immune senescence and increase the ability of older animals to fight *Salmonella* infections. *In vitro* studies showed that replicative senescence was reversed by LFU, and senescence markers were decreased in organs of aged mice after LFU treatment (14). Thus, restoration of the immune system fits with the general rejuvenation after LFU treatment.

## MATERIALS AND METHODS

### Bacterial strains and growth conditions

For infections, *Salmonella enterica* serovar Typhimurium strain SL1344 was used to inoculate mice. A single bacterial colony was grown in 3 mL Luria Bertani (LB) broth overnight at 37°C with shaking at 250 rpm (SPI-1 inducing conditions). The next day, 100 µL of the overnight culture was sub-cultured into 3 mL LB broth and incubated at 37°C with shaking at 250 rpm for 5 h until the optical density at 600 nm (OD_600_) reached ~2.5.

### Animals and infections

C57BL/6J male mice were purchased from Jackson Laboratory, and young mice (4–6 months) and old mice (21–24 months) were used according to Institutional Animal Care and Use Committee (IACUC) protocol #1910084A. Animal weights were measured daily, and animals were euthanized after loss of >20% of their initial body weight. Animals were pre-treated with streptomycin (Sigma) as follows: food and water were removed for 4 h before oral gavage of 20 mg streptomycin in 150 µL sterile water (16). The next day, food and water were removed for 4 h before oral gavage of 1 × 10^6^ *Salmonella* in 200 µL Phosphate buffered saline (PBS) per mouse. The animals were monitored for 3 days and then euthanized with CO_2_ asphyxiation followed by cervical dislocation. The livers, spleens, and blood were collected according to published protocols (17). The blood serum was immediately prepared by leaving the blood undisturbed to clot at Room Temperature (RT) for 30 min, followed by centrifugation at 2,000 × g for 10 min in a refrigerated centrifuge. After euthanasia, the livers and spleens were collected in sterile PBS and homogenized using an Omni tissue homogenizer, and dilutions were plated on LB agar with streptomycin (100 µg/mL). The number of bacteria in each organ was reported as CFU per gram of tissue.

### LFU treatment

Prior to infection, one group of old mice was treated with LFU for 30 min every other day for 6–9 times. The treatment was performed following LFU procedures described in Kureel et al. (14).

### Colonization of livers and spleens

After euthanasia, the livers and spleens were collected in sterile PBS and homogenized using a tissue tearer (Omni tissue homogenizer), and dilutions were plated on LB agar with streptomycin (100 µg/mL). The number of bacteria in each organ was reported as CFU per gram of tissue.

### Blood analysis

Whole blood analysis was performed by a fully automated Blood Cell analyzer (Zoetis, HM5) following the manufacturer’s manual using whole blood collected in tubes containing potassium EDTA (purple/lavender top). The reported number of blood cells was plotted for each mice group.

### Cytokine analysis

The proteome profiler mouse cytokine array (#ARY006, R&D systems) was analyzed according to the manufacturer’s instructions using blood serum. The level of cytokines in each group was measured as mean pixel density using ImageJ software.

## RESULTS AND DISCUSSION

### LFU treatment reduced susceptibility to *Salmonella* infection in aged mice

**Fig 1:**
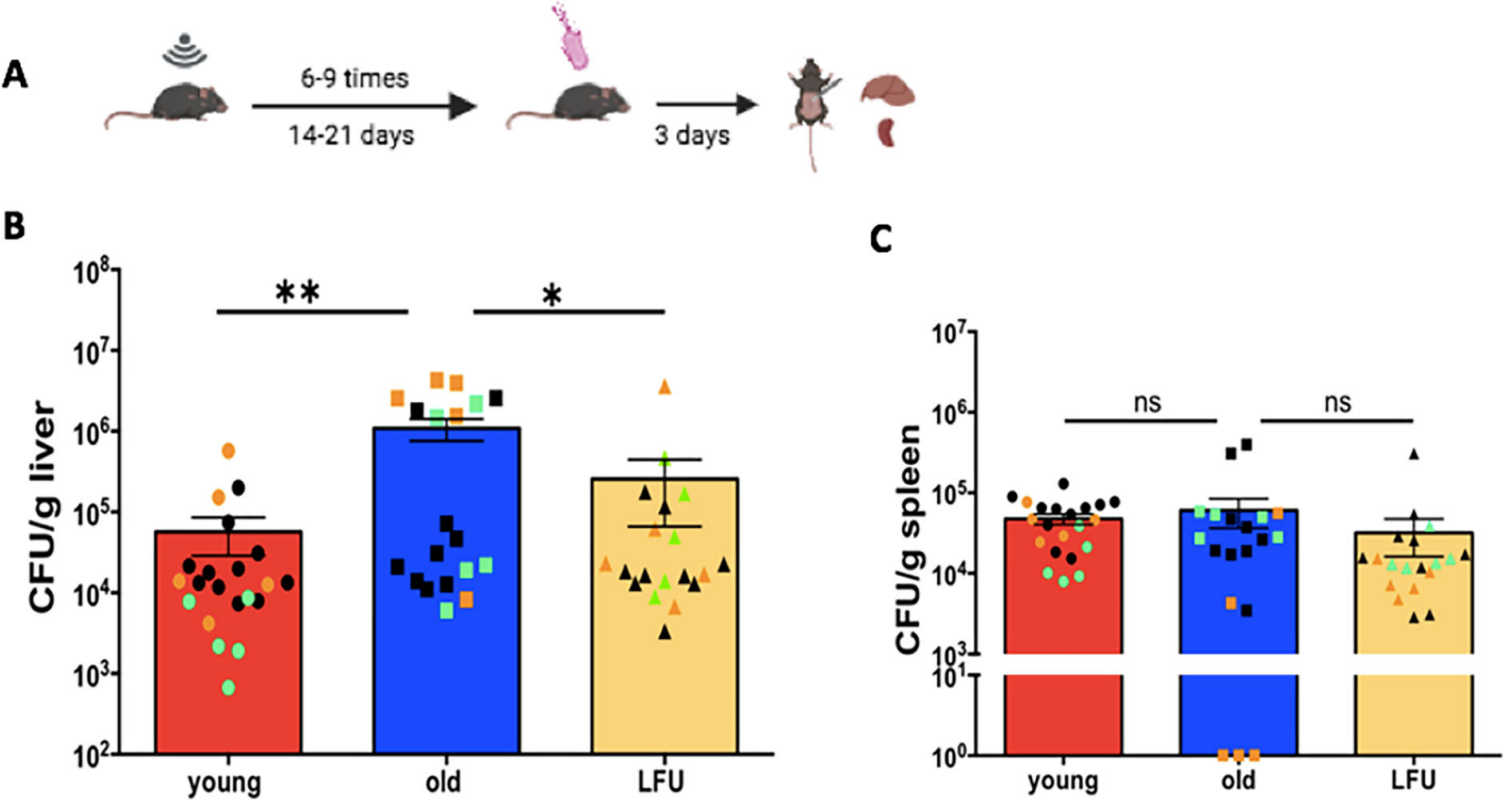
LFU treatment prior to infection reduces the *Salmonella* burden in the livers of aged mice at 3 dpi. One group of C57BL/6J young male mice aged 4–6 months and two groups of C57BL/6J old male mice aged 21–24 months were orally infected with *Salmonella* Typhimurium. Prior to infection, one group of old mice received LFU treatment for 30 min every other day for 2–3 weeks (6–9 treatments). Bacteria recovered from the livers was calculated as CFU per gram of tissue. (**A**) a graphic description of the experimental procedure. (**B**) The number of bacteria recovered from livers and (**C**) spleens. The number of bacteria recovered from the livers of old mice was 19-fold higher than the number of bacteria recovered from the livers of young mice. LFU treatment of old mice prior to *Salmonella* infection resulted in reduced susceptibility to infection, as indicated by the lower CFU recovered from the livers compared to old, untreated mice. Red bar, young untreated mice; blue bar, old untreated mice; yellow bar, old LFU-treated mice. Data are mean ± SEM (*n* = 19–21 mice per group) from three independent experiments (shown by orange, green, and black symbols). Statistical significance using Student’s *t*-test. *P value < 0.05 and **P value < 0.001.

One group of old mice received LFU treatment every other day for 6–9 times prior to infection as described in reference 14. The control mice (one group each of young mice or old mice) were placed in the LFU bath with no additional treatment. All mice were then infected with *Salmonella* by oral gavage and euthanized 3 days post-infection (3 dpi). The number of bacteria in their livers was determined and is plotted in Fig. 1A. The average number of bacteria in the livers of old mice (1.1 × 10^6^) was ~19 times higher than the average number of bacteria in the livers of young mice (5.7 × 10^4^), indicating an increased susceptibility of aged animals to *Salmonella* infection (Fig. 1B). This observation was consistent with earlier reports in which CFU in the livers of old mice (3) and old rats (2) were higher than young mice and rats. Interestingly, pre-treatment of old mice with LFU reduced the CFU burden of bacteria in the liver (2.6 × 10^5^) by about fourfold compared to old, untreated mice (1.1 × 10^6^; Fig. 1B). The reduced number of bacteria in the livers of old LFU-treated mice suggested that LFU pre-treatment had a rejuvenating effect by reducing the susceptibility of old mice to *Salmonella* infection.

We next examined the effect of LFU treatment on the CFU of bacteria recovered from spleens and measured the spleen weight in the three groups of mice after 3 dpi. The CFU of bacteria in the spleens was not significantly different between the three groups of mice at 3 dpi. The average CFU of bacteria in the spleens of old mice (6.1 × 10^5^) was slightly higher (~1.3) than the average CFU in the spleens of young mice (4.8 × 10^5^) (Fig. 1C), consistent with earlier reports from rats (2). The average CFU of bacteria in the spleen of old mice that were treated with LFU was approximately twofold lower than old, untreated mice (3.2 × 10^5^ compared to 6.1 × 10^5^, respectively; Fig. 1C), although these differences were not significant. The splenic levels of bacteria were only slightly greater with age; however, the LFU-treated older animals still experienced a reduction in bacterial burden.

### LFU had no effect on weight loss

**Fig 2:**
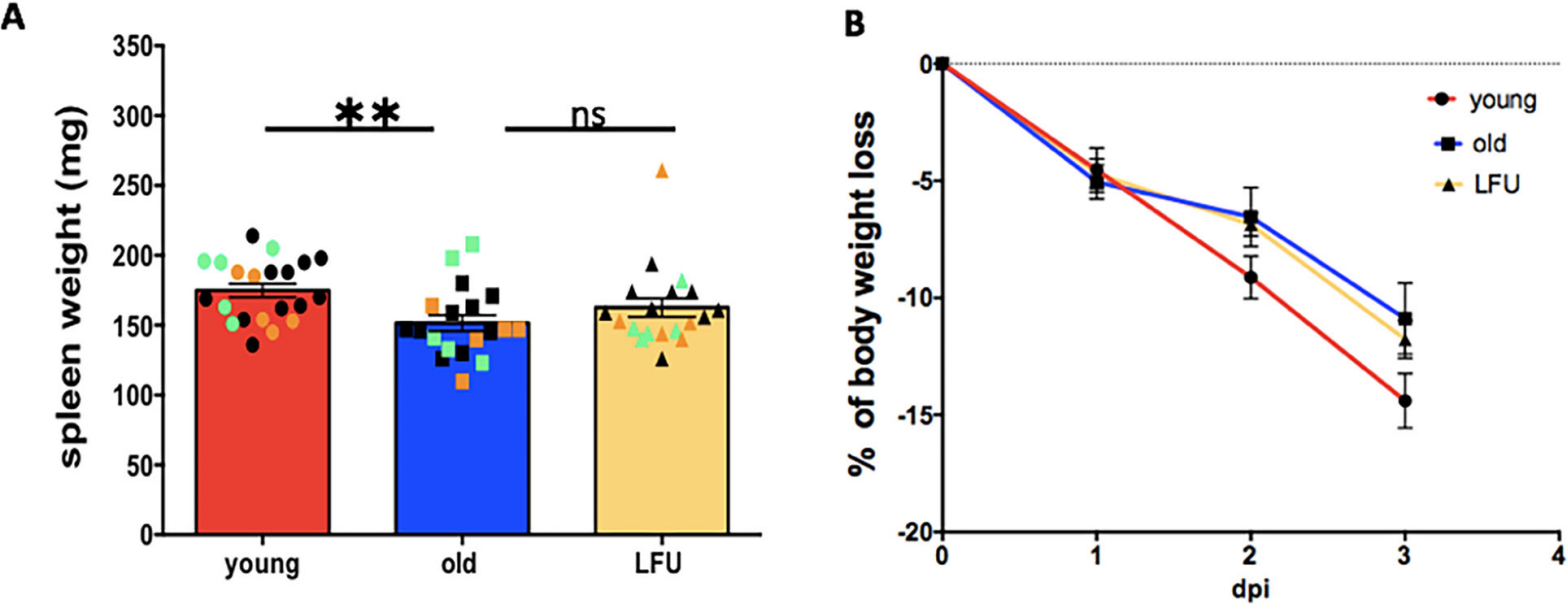
LFU has no effect on the weight of old mice infected with *Salmonella* Typhimurium. (**A**) Spleen weight. (**B**) Percentage of body weight lost in young, old, and old LFU-treated mice. Red bar, young untreated mice; blue bar, old untreated mice; and yellow bar, old LFU-treated mice. Data are mean ± SEM (*n* = 19–21 mice per group) from three independent experiments (shown by orange, green, and black symbols). Statistical significance using Student’s *t*-test. *P value < 0.05 and **P value < 0.001.

The average spleen weight of infected old mice was significantly reduced by about 13% compared to that of young mice (151.5 mg vs 175 mg; Fig. 2A). It was also approximately 7% lower than that of old LFU-treated mice (163 mg). Spleen “shrinkage” in infected old mice might be due to the smaller number of splenocytes (17, 18). We next examined the effect of LFU on weight loss during the infection period (Fig. 2B). The average percentage of weight loss in old mice was slightly higher (5%) compared to young mice (4.5%) and old LFU-treated mice (4.7%) at 1 dpi (Fig. 2B). However, the young mice lost more weight (14.4%) compared to untreated (11%) and LFU-treated (11.8%) old mice by 3 dpi. This finding contradicts an earlier report, which could be due to high variability in different batches of aging mice (3).

### LFU does not affect blood profiles

**Fig 3:**
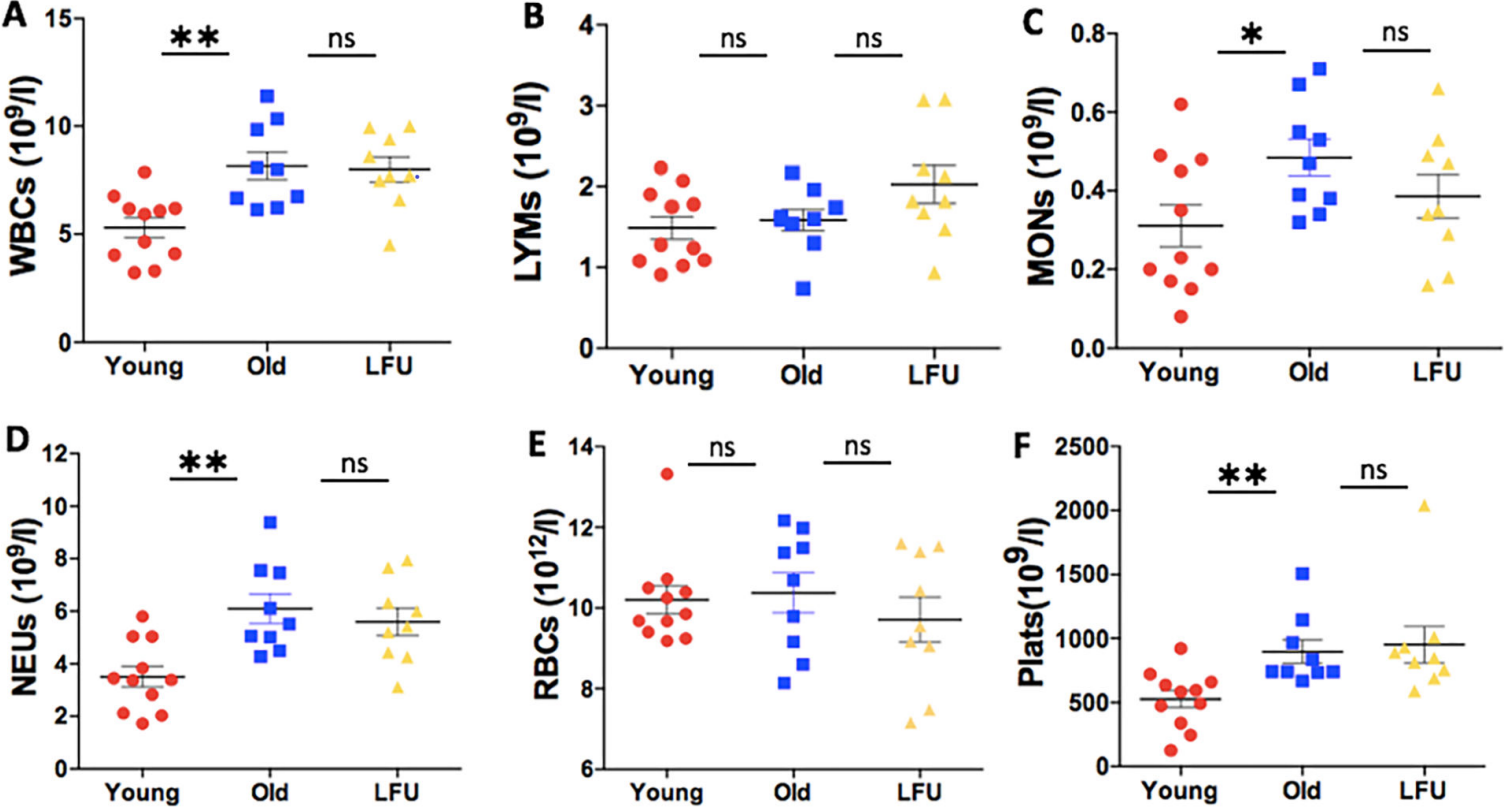
LFU has no effect on the blood profiles of old mice infected with *Salmonella* Typhimurium at 3 dpi. The total number of (**A**) WBCs, (**B**) lymphocytes, (**C**) monocytes, (**D**) neutrophils, (**E**) RBCs, and (**F**) platelets in young, old, and old LFU-treated mice. Red, young untreated mice; blue, old untreated mice; yellow, old LFU-treated mice (*n* = 9–11 mice per group). The data are presented as the mean ± SEM. Statistical significance using Student’s *t*-test. *P value < 0.05 and **P value < 0.001; ns = not significant.

We next analyzed the blood profiles of mice after 3 dpi (Fig. 3). The number of blood cells was plotted for each group of mice. Blood analysis determined the number of white blood cells (WBCs), lymphocytes, monocytes, neutrophils, red blood cells (RBCs), and platelets. The number of lymphocytes and RBCs was not different in young mice compared to old mice. WBCs, monocytes, neutrophils, and platelets were all higher in old mice compared to young mice, and LFU treatment did not affect these numbers. An increase in the number of neutrophils and macrophages in older mice was reported previously (3). They speculated that the inability of old mice to induce youthful levels of neutrophils upon *Salmonella* infection, along with lower levels of TNF-α, resulted in a higher susceptibility to infection in aged mice.

### LFU effects on chemokine profiles at 3 dpi

**Fig 4:**
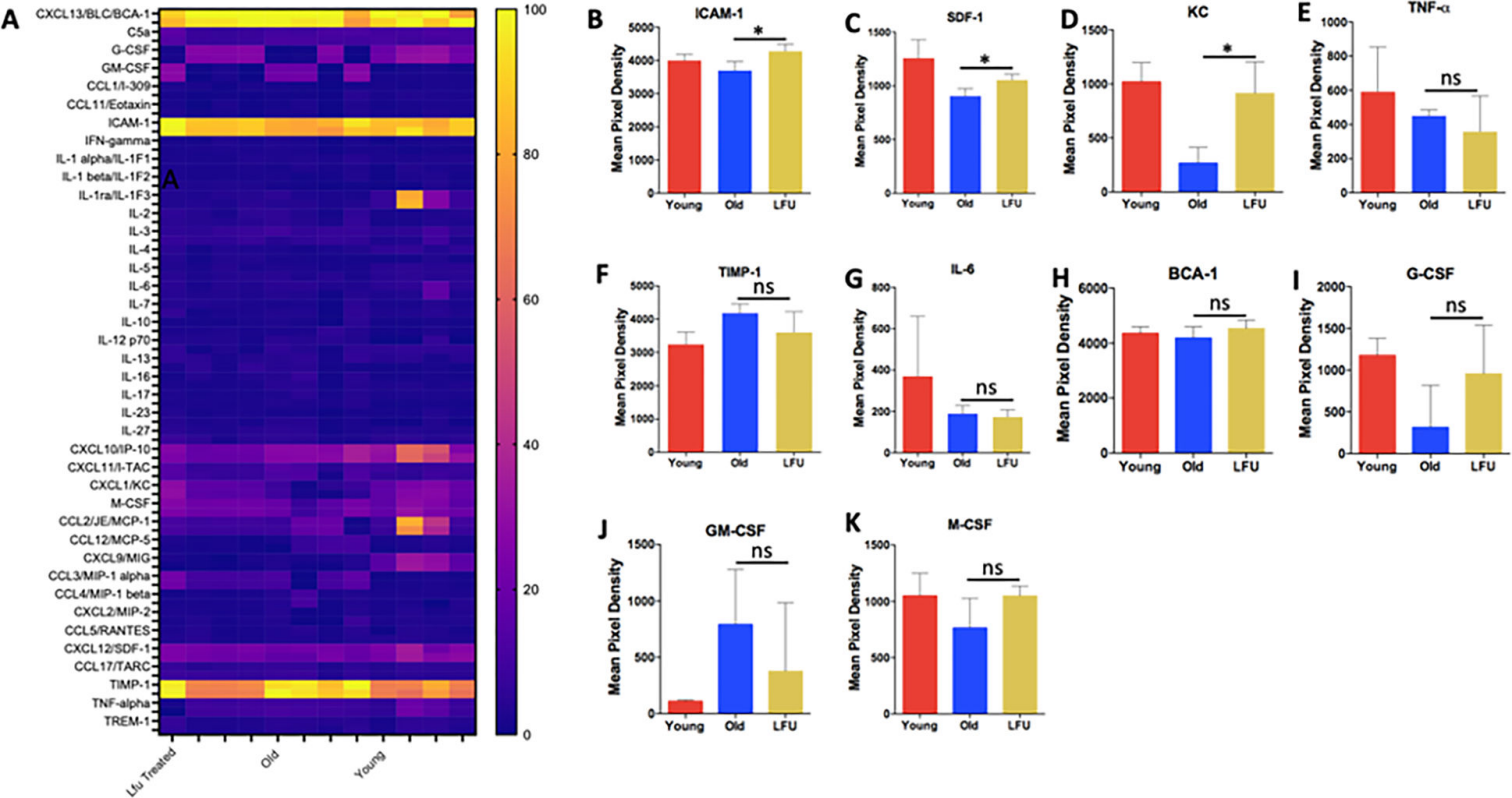
LFU effects on chemokine profiles of old mice infected with *Salmonella* Typhimurium at 3 dpi. The level of chemokine/cytokine (**A**) heat map representing the differential level of chemokines between three groups of infected young, old, and LFU-treated old mice. The hatch marks are four biological replicates in each group. The 0–100 scale represents the normalized expression value for each data point. Zero percent typically corresponds to the minimum value in the data set, and 100% corresponds to the maximum value, with intermediate values mapped linearly between them, making it easier to visually compare trends across different data sets or conditions. (**B**) ICAM-1, (**C**) SDF-1, (**D**) KC/CXCL1, (**E**) TNF-⍺, (**F**) TIMP-1, (**G**) IL-6, (**H**) BCA-1, (**I**) granulocyte colony-stimulating factor (G-SCF), (**J**) granuolocyte-macrophage colony-stimulating factor (GM-CSF), and (**K**) macrophage colony-stimulating factor (M-CSF) levels in infected young, old, and old LFU-treated mice are presented as mean pixel density. TIMP-1 and GM-CSF levels increased in aged mice compared to young mice. LFU pre-treatment had no significant effect. In contrast, the levels of TNF-α, IL-6, BCA-1, G-CSF, and M-CSF were lower in old mice compared to young mice, and LFU treatment had no significant effect. Red bar, young untreated mice; blue bar, old untreated mice; and yellow bar, old LFU-treated mice (*n* = 9–11 mice per group). The data are presented as the mean ± SEM. Statistical significance using Student’s *t*-test. *P value < 0.05.

**Fig 5:**
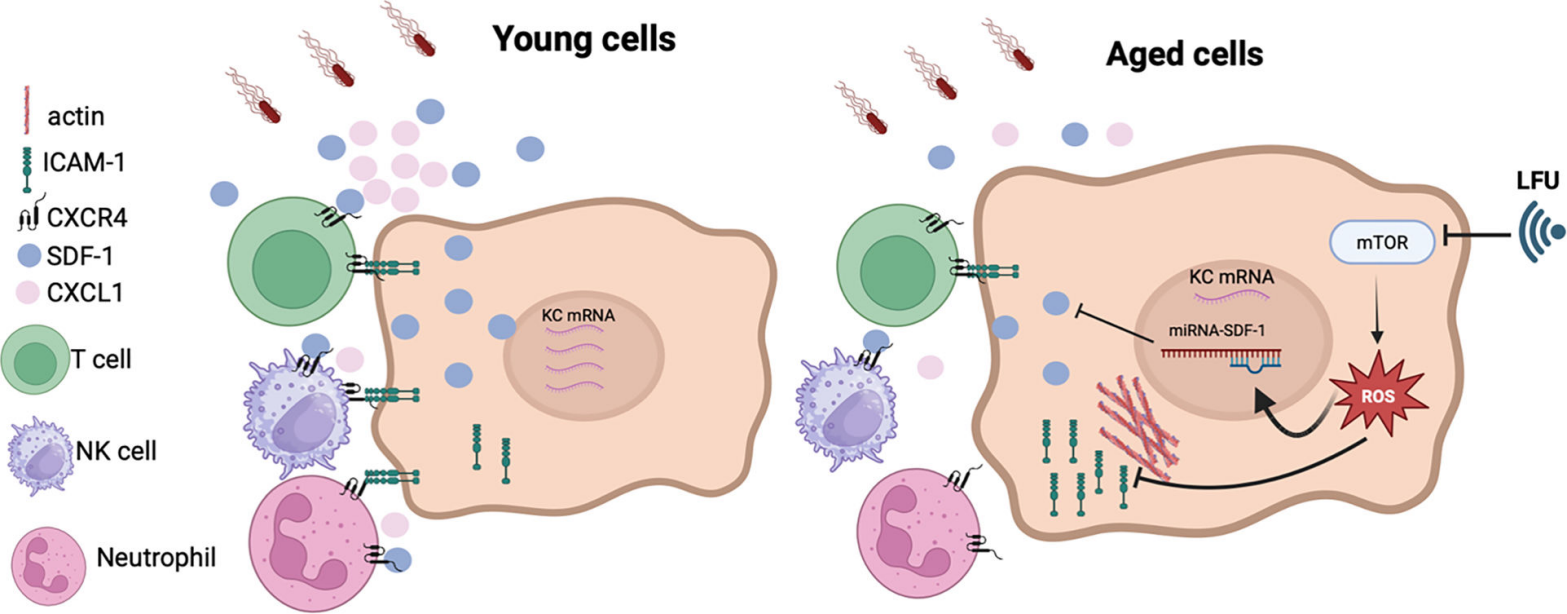
LFU impacts immune senescence, reducing susceptibility to *Salmonella* infection in aged cells. Upon *Salmonella* infection (**A**), young cells produce functional levels of SDF-1, KC/CXCL1, and ICAM-1, recruiting immune cells to the infection site. In aged cells (**B**), due to high ROS production, miRNA-141 suppresses SDF-1 production, as well as reduces KC/CXCL1 expression and inhibits the interaction of ICAM-1 with actinin, blocking the transport of ICAM-1 to the cell surface. The reduction in SDF-1, KC/CXCL1, and ICAM-1 at the cell surface reduces immune cell attraction to the site of infection, increasing susceptibility to infection in aged cells. LFU blocks mTOR and reduces ROS (14), rejuvenating aged cells by increasing chemokine production in response to *Salmonella* infection. Generated using BioRender.com.

To understand the effect of LFU treatment, we next analyzed chemokine and cytokine levels in young, old, and old LFU-treated mice. The heat map revealed induction of B cell-attracting chemokine 1 (BCA-1), Intracellular Adhesion Molecule 1 (ICAM-1), and Tissue Inhibitor of Metalloproteinase 1 (TIMP-1) upon infection in all three groups (Fig. 4A). However, further analysis of individual chemokines revealed some important differences. The levels of ICAM-1 and Stromal Cell-Derived Factor 1 (SDF-1) and Cytokine-induced neutrophil chemoattractant (KC/CXCL1) were reduced in aged mice compared to young mice (Fig. 4B through D). LFU treatment led to a significant increase in these chemokines in aged mice, restoring them to levels observed in young mice. Interestingly, ICAM-1, SDF-1, and KC/CXCL1 are age-dependent chemokines involved in attracting immune cells to the site of infection. Decreased interaction of ICAM-1 with actinin in aged cells results in reduced transport of ICAM-1 to the cell membrane (see Model, Fig. 5). SDF-1 mediates attraction of natural killer cells, T cells, and neutrophils to the infection site (19). Production of reactive oxygen species (ROS) in aged cells led to activation of miRNA-141 (20), which suppressed SDF-1 in aged mice and human bone marrow (21). KC/CXCL1 attracts neutrophils *in vitro* and *in vivo*. In splenic B cells isolated from aged mice, KC/CXCL1 expression was lower than in young mice after lipopolysaccharide (LPS) activation. The weaker aged B cells recruited fewer leukocytes to infection sites (22). In previous studies, LFU reduced ROS and inhibited mammalian target of rapamycin (mTOR) in aged cells (14). This would activate SDF-1 and KC/CXCL1 production and increase ICAM-1 transport to the cell surface, attracting more immune cells to the infection site and reducing the organ burden of *Salmonella* in aged mice (Fig. 5).

### Summary

In conclusion, our study demonstrates the efficacy of LFU treatment in significantly rejuvenating the immune system in aged mice and reducing their susceptibility to *Salmonella* infection. These findings underscore the potential of LFU as a therapeutic intervention to boost immune function in elderly populations, reducing the risk of infectious diseases. It is interesting to note that recent studies on LFU effects on diabetes and insulin resistance induced in mice fed a high-fat, high-sucrose diet and treated with streptozotocin also reported that LFU treatment reduced inflammation and altered immune cell function in skeletal muscle in insulin-resistant and diabetic populations (15). Further experiments are focused on the development of this non-invasive approach for the treatment of numerous age-related maladies.
